# Comment on “A Pumpless Microfluidic Neonatal Lung Assist Device for Support of Preterm Neonates in Respiratory Distress”

**DOI:** 10.1002/advs.202004382

**Published:** 2021-05-03

**Authors:** Li Wang, Fang Li, Zhichun Feng, Yuan Shi

**Affiliations:** ^1^ Department of Pediatrics University‐Town Hospital of Chongqing Medical University Chongqing 401331 China; ^2^ Department of Pediatrics Daping Hospital Army Medical University Chongqing 400042 China; ^3^ Department of Neonatology Ministry of Education Key Laboratory of Child Development and Disorders National Clinical Research Center for Child Health and Disorders China International Science and Technology Cooperation Base of Child Development and Critical Disorders Children's Hospital of Chongqing Medical University Chongqing Key Laboratory of Pediatrics Chongqing 400014 China; ^4^ Affiliated BaYi Children's Hospital General Hospital of the People's Liberation Army Beijing 100007 China

**Keywords:** lung assist device, neonates, respiratory distress

Very recently, Dabaghi et al. operated a microfluidic, artificial placenta‐type neonatal lung assist device (LAD) on a newborn piglet with respiratory distress.^[^ ^1^ ^]^ Results from the piglet experiments revealed the effectiveness of this LAD in gas exchange without complications. The authors indicated that the LAD has a potential application as a biomimetic artificial placenta to support the respiratory needs of preterm neonates. We consider their study findings to be an incredibly important contribution to reduce the mortality of premature infants, especially those born earlier than 28 weeks of gestational age.

As we know, mechanical ventilation is often used for respiratory support of neonates with respiratory failure. However, it is invasive and associated with severe complications such as pulmonary injury, chronic lung disease, and related diseases of prematurity such as retinopathy of prematurity, intraventricular hemorrhage, or necrotizing enterocolitis, which would lead to several long‐term side effects.^[^ ^2^, ^3^ ^]^ In late‐preterm and term infants, extracorporeal membrane oxygenation (ECMO) could be an alternative choice of treatment, but ECMO requires central vascular access by surgery, as well as their high priming volume and external pump for perfusion are not suitable for neonatal infants.^[^ ^4^ ^]^ Through the experiment by Dabaghi et al.,^[^ ^1^ ^]^ we learned that LAD has the characteristics of high‐performance, and pumpless. In addition, an important element of LAD is that the newborn will continue to breathe while the LAD provides additional gas exchange and allows the lungs to heal. In this way, some complications and limitations due to performing mechanical ventilation or ECMO could be prevented. Therefore, we believe that the LAD has very important clinical application value and prospect in newborn infants with respiratory failure.

After reading the article carefully, we have some questions to discuss with authors and other neonatologist.

First, one of the characteristics of the microfluidic device reported by the authors is pumpless extracorporeal support, which uses the pressure generated solely by the piglet's heart to promote blood circulation. We noticed that, at this developmental stage of experiment, authors still used the vascular access via carotid artery and jugular vein that is near the heart and is helpful to promote the blood circulation by the pressure difference produced by the heart. However, the concept of the artificial placenta is designed to be connected to the umbilical vessels. We would, therefore, like to enquire how could this kind of pumpless device ensure the proper flow rate to fully oxygenate the blood if the umbilical cord is connected according to the author's idea in the further step, whether the research team has conducted relevant experimental evaluation?

Second, although the hydraulic resistance of the oxygenator can be customized according to the baby's weight to generate sufficient blood perfusion and oxygen uptake, with the increase of neonatal weight and the remission of lung disease, the heart's pumping capacity will change, which will inevitably affect the blood flow rate and oxygen supply of LAD circulation. How to adjust it in real time? Increase or decrease the number of microfluidic blood oxygenators (MBOs)? Is it difficult to adjust accurately?

Third, Dabaghi et al. chose to use the vascular access via carotid artery and jugular vein as they found the umbilical vessels in newborn piglets were fragile and will shrink within a few hours after birth, so the catheters used were small and could not reach the intended flow rate of 30 mL kg^–1^ min^–1^ when LAD was connected to the umbilical vessels in the piglet model.^[^ ^5^ ^]^ Moreover, according to the previous experience of umbilical catheterization in newborn infants, the time for umbilical cord to retain function and reduce the risk of infection after birth is limited. In the limited time, it could not ensure that the lung development of premature infants is mature enough to leave the artificial placenta for oxygen supply. How to solve these problems?

In addition, it is well known that fetal circulation should be transferred to postnatal circulation after birth. The LAD device is expected to connect to umbilical cord for oxygen supply. Have authors assessed whether the retention of fetal circulation will affect the decrease of pulmonary circulation pressure and the expansion of alveoli?

Finally, authors described in the original article that the LAD was exposed to pure oxygen and air and the blood flow through the LAD loop, but it was not described in detail whether the device of artificial placenta/MBOs is in a relatively closed space or some necessary measures have been taken to prevent infection. Could the author specify?

In conclusion, respiratory support technology plays an important role in reducing the mortality of newborns, especially premature infants. From mechanical ventilation, ECMO, to artificial placenta, it has witnessed the continuous improvement of neonatal respiratory support technology. We appreciate the authors’ excellent research work, from in vitro experiments, to in vivo experiments on newborn lambs, and to in vivo experiments on newborn piglets (of similar size to neonates). However, there are still many details to be discussed and improved in the application of LAD to newborns with respiratory failure. We hope that more neonatologists could pay attention to this great technology and participate in its development, and we expect LAD could be used clinically as soon to help those preterm infants with respiratory failure.

## Author Contribution

L.W. and F.L. contributed equally to this work.

## Conflict of Interest

The authors declare no conflict of interest.

## Data Availability

Data sharing is not applicable to this article as no new data were created or analyzed in this study.

## References

[1] M. Dabaghi , N. Rochow , N. Saraei , G. Fusch , S. Monkman , K. Da , A. Shahin‐Shamsabadi , J. L. Brash , D. Predescu , K. Delaney , C. Fusch , P. R. Selvaganapathy , Adv. Sci. 2020, 7, 2001860. 10.1002/advs.202001860 PMC7610273 33173732

[2] A. G. S. Philip , Neonatology 2012, 102, 1. 22354063 10.1159/000336030

[3] E. A. Jensen , S. B. DeMauro , M. Kornhauser , Z. H. Aghai , J. S. Greenspan , K. C. Dysart , JAMA Pediatr. 2015, 169, 1011. 26414549 10.1001/jamapediatrics.2015.2401 PMC6445387

[4] A. F. J. van Heijst , A. C. de Mol , H. Ijsselstijn , Semin. Perinatol. 2014, 38, 104. 24580766 10.1053/j.semperi.2013.11.008

[5] N. Rochow , A. Manan , W.‐I. Wu , G. Fusch , S. Monkman , J. Leung , E. Chan , D. Nagpal , D. Predescu , J. Brash , P. R. Selvaganapathy , C. Fusch , Artif. Organs 2014, 38, 856. 24716531 10.1111/aor.12269

